# BMX-ARHGAP fusion protein maintains the tumorigenicity of gastric cancer stem cells by activating the JAK/STAT3 signaling pathway

**DOI:** 10.1186/s12935-019-0847-5

**Published:** 2019-05-17

**Authors:** Xiao-Feng Xu, Feng Gao, Jian-Jiang Wang, Cong Long, Xing Chen, Lan Tao, Liu Yang, Li Ding, Yong Ji

**Affiliations:** 1 Clinical Laboratory, Jingjiang People’s Hospital, Jingjiang, 214500 People’s Republic of China; 2 Department of Surgery, Jingjiang People’s Hospital, No. 28, Zhongzhou Road, Jingjiang, 214500 Jiangsu People’s Republic of China; 3 Central Laboratory, Jingjiang People’s Hospital, Jingjiang, 214500 People’s Republic of China

**Keywords:** BMX-ARHGAP, Src homology region 2, Gastric cancer, JAK/STAT3 signaling pathway, Cancer stem cells

## Abstract

**Background:**

Cancer stem cells (CSCs), drug-resistant cancer cell subsets, are known to be responsible for tumor metastasis and relapse. The JAK/STAT pathway, activated by SH2 domain, is known to regulate the tumor growth in gastric cancer (GC). Now, this study was designed to examine whether BMX-ARHGAP affects the GC stem cell properties and the underlying regulatory network via JAK/STAT axis.

**Methods:**

BMX-ARHGAP expression was characterized in GC tissues and cells by RT-qPCR and western blot assay. When BMX-ARHGAP was overexpressed or silenced via plasmids or siRNA transfection, the stem cell properties were assessed by determining stem cell markers CD133, CD44, SOX2 and Nanog, followed by cell sphere and colony formation assays. Subsequently, cell proliferation and invasion were examined by conducting EdU and Transwell assays. The JAK/STAT3 signaling pathway activation was inhibited using AG490. ARHGAP12, BMX exon 10–11, BXM-SH2, JAK2 and STAT3 expression patterns were all determined to examine the regulatory network. The stem cell property in nude mice was also tested.

**Results:**

BMX-ARHGAP was determined to be enriched in the GC. Overexpression of BMX-ARHGAP resulted in increased expression of CD133, CD44, SOX2 and Nanog protein, and accelerated proliferation and invasion of CD133^+^CD44^+^ cells as well as facilitated self-renewal potential of GC cells. However, the inhibition of the JAK/STAT3 signaling pathway reversed the stimulating effect of BMX-ARHGAP on proliferative and invasion abilities of CD133^+^CD44^+^ cells. The overexpression of BMX-ARHGAP was suggested to increase the BMX-SH2 protein expression via ARHGAP 5′UTR, and activate the JAK/STAT3 signaling pathway. Also, BMX-ARHGAP promoted tumor growth in nude mice.

**Conclusions:**

The aforementioned results demonstrated that the BMX-ARHGAP-dependent SH2 domain-JAK/STAT3 axis mediates the maintenance of GC stem cells, benefiting the development of new potential therapeutic targets for GC.

## Background

Gastric cancer (GC) ranks as the leading cause of cancer-related deaths in China, with a poor prognosis and poor 5-year survival rate resulting from a delayed diagnosis at an advanced stage [1]. Due to the present circumstances, surgery is the only curative strategy, while either adjuvant or neoadjuvant therapy is generally recommended for locally advanced GC, not at an end stage [2]. Although the morbidity of GC is decreasing, the stage IV metastatic GC at primary diagnosis shows an incurable outcome coupled with a rather poor prognosis [3]. Cancer stem cells (CSCs), characterized by multi-potency, high self-renewal, and tumorigenic capacities, are all proposed to be associated with cancer maintenance, metastasis, relapse, and drug resistance [4, 5]. A variety of gene mutations could induce cancer progression, and the accurate identification of these genes is conducive to the development of novel target drugs and treatments [6]. Strikingly enough, one of these genetic alteration (fusion genes), also known as chimeric genes formed in cancers, are potential good candidates for molecular targets in the treatment of cancers [7]. Gene fusions with oncogenic properties could be triggered by specific genomic aberrations including translocations, deletions, amplifications, and genome rearrangements [8, 9]. Hence, the identification of novel gene fusion and its mechanism in cancer progression have great value in developing specifically targeted therapies.

A novel gene fusion, BMX-ARHGAP has been previously identified in gastric cardia adenocarcinoma [10]. Bone marrow kinase on chromosome X (BMX) has been demonstrated to be expressed in several cancers, and to modulate the survival and tumorigenicity of cancer stem cells involved in glioblastoma [11]. BMX is suggested to function as a tumor promoter, inhibiting chemotherapy- and radiotherapy-induced apoptosis in colorectal cancer [12]. RhoA GTPase activating protein (ARHGAP) family contains a series of cancer-associated proteins [13]. For example, ARHGAP18 has been demonstrated to suppress cancer progression and tumor growth in GC [14]. More importantly, the suppression of BMX-ARHGAP was suggested to restrain GC progression through the blockade of the Janus kinase (JAK)-signal transducer and activator of transcription (STAT) signaling pathway [15]. JAK/STAT signaling pathway is implicated in the tumorigenesis of GC [16]. The inhibition of the JAK2/STAT3 signaling pathway has also been reported to participate in the suppression of angiogenesis and metastasis in hepatocellular carcinoma [17]. Src-homology 2 (SH2)-domain binds phosphorylated tyrosine residue, regulating the roles of intracellular receptor signal transduction pathways in cancer progression including the JAK/STAT signaling [18, 19]. Based on these findings, we speculated that BMX-ARHGAP fusion might regulate SH2-domain containing protein, while further mediating the GC progression via the JAK/STAT3 signaling pathway. In the following experiments, we made efforts in examining the regulatory effects that BMX-ARHGAP had on the GC stem cell markers and their properties, and analyzing the potential regulatory network of BMX-ARHGAP/SH2/JAK/STAT3 to provide an anti-cancer target for cancer treatment.

## Materials and methods

### Ethics statement

The patient study was conducted with the general approval of the ethic committee of Jingjiang People's Hospital and in accordance with the Declaration of Helsinki statement: ethical principles for medical research involving human beings. Written informed consent forms were signed and obtained from all subjects or their guardians. The animal experiments were carried out in strict accordance with the recommendations provided by the management and use of Laboratory Animals promulgated by the National Institutes of Health.

### Study subject

GC tissues and adjacent normal tissues were surgically removed from 165 patients who had been diagnosed with GC at the Jingjiang People's Hospital from March, 2015 to June, 2018, and preserved at − 80 °C. The surveyed patient pool consisted of 109 males and 56 females with a mean age of 65.45 ± 7.68 years. The gastroscopy and pathological examination confirmed the diagnosis of GC. The following cases had been excluded: the patients suffering from severe malnutrition, other tumors or dysfunction of important organs such as heart and lung; patients who had been administered with any immune-suppressor, hormone drugs, or blood products; patients who had received any form of cancer treatment such as chemoradiotherapy, immunotherapy or other therapies prior to surgery.

### Immunohistochemistry

Paraffin-embedded GC or adjacent normal tissue samples were sliced into sections, followed by dehydration with gradient ethanol. Next, the tissue sections were treated with 3% H_2_O_2_ in methyl alcohol for 20 min, washed under distilled water for 2 min, followed by a washing with 0.1 M phosphate buffer saline (PBS) for 3 min in sequence, ending with an antigen retrieval in a water bath. The tissue sections were incubated with primary antibodies to SOX2 (ab97959, 1:1000), p-JAK2 (ab32101, 1:1000), Nanog (ab80892, 1:400), p-STAT3 (ab76315, 1:200) overnight at room temperature. The antibodies used in this assay were all from Abcam, Inc. (Cambridge, MA, USA). Then, the tissue sections were incubated with the secondary antibody, and goat anti-rabbit antibody to immunoglobulin G (IgG) (ab6785, 1:1000) at 37 °C for 30 min. Subsequently, the sections were stained using a horseradish peroxidase (HRP)-labeled streptavidin (0343-10000U, Yimo biotechnology Co., Ltd., Beijing, China) at 37 °C for 20 min, followed by another PBS washing (5 min × 3 times). Following, the sections were stained by diaminobenzidine (DAB, ST033, WHIGA technology Co., Ltd., Guangzhou, Guangdong, China), and counterstained by hematoxylin for 1 min. The sections were differentiated using a hydrochloric ethanol and subsequently washed under tap water. After dehydration and deparaffinizing, the tissue sections were mounted with neutral resin. The staining results were observed among 5 randomly selected fields under a microscope, with one hundred cells being calculated for each field. Each experiment was conducted a total of 3 times.

### Cell line selection and cell transfection

Four human GC cell lines (SNU-5, MNK-45, AGS and SGC7901) and the normal gastric epithelial cell line (GES-1) were purchased from American Type Culture Collection (Manassas, VA, USA). The cells were cultured in Royal Park Memorial Institute (RPMI) 1640 (Gibco, Carlsbad, CA, USA) supplemented with a 10% fetal bovine serum (Gibco, Carlsbad, CA, USA), 100 μg/mL streptomycin, and 100 U/mL penicillin in a 5% CO_2_ incubator at 37 °C (Thermo Fisher Scientific, Waltham, MA, USA). The expression of BMX-ARHGAP in each cell line was determined by reverse transcription quantitative polymerase chain reaction (RT-qPCR). SGC7901 cells with highest expression of BMX-ARHGAP was employed for further experimentation.

The SGC7901 cells in the logarithmic growth phase were detached using trypsin, and then cultured in a 6-well plate at a density of 1 × 10^5^ cells for 24 h. The scramble shRNA (sh-NC, 5-GGGTGAACTCACGTCAGAA-3), shRNA targeting BMX-ARHGAP (sh-BMX-ARHGAP, 5-GAUCACAAUCUGAACAGUUACUCAG-3), pcDNA3.1, and BMX-ARHGAP overexpression (pcDNA3.1-BMX-ARHGAP) plasmids were all constructed according to a previous literature [15]. When the cell confluence reached approximately 75%, the cells were transfected with the aforementioned plasmids (50 ng/mL) using Lipofectamine 2000 (Invitrogen, Carlsbad, CA, USA). The transfected cells were then exposed to G418 (300 mg/L) to effectively screen the cell clones. AG490 was utilized as a specific inhibitor for the JAK/STAT3 signaling pathway, and PBS served as a control.

The protein expression of BMX-ARHGAP, BMX and ARHGAP in the successfully transfected cells was determined by conducting a western blot assay.

### RNA extraction and quantification

Total RNA was extracted using a Trizol kit (15596026, Invitrogen, Carlsbad, CA, USA), and subsequently reversely transcribed into cDNA using a Prime Script RT reagent Kit (RR047A, Takara, Tokyo, Japan). Then, a real time quantitative PCR was conducted using Fast SYBR Green PCR kit (Applied biosystems, Foster City, CA, USA), on an ABI7500 real-time quantitative PCR instrument (Applied biosystems, Foster City, CA, USA). The reaction system (20 µL) consisted of 10.0 µL of 2× SYBR Green, 0.3 µL of forward primer (20 µM), 0.3 µL of reversed primer (20 µM), 1.0 µL of cDNA, and 8.4 µL of RNase Free ddH_2_O. The reaction condition was set as the following: pre-denaturation at 95 °C for 5 min, 40 cycles of denaturation at 95 °C for 30 s, annealing at 60 °C for 1 min. Moreover, 3 replicates were established for each well. All primers are shown in Table 1, with glyceraldehyde-3-phosphate dehydrogenase (GAPDH) used as an internal reference. The relative expression of target gene was calculated using the 2^−ΔΔCt^ method: ΔΔCt = Ct (target gene_the experimental group_ − internal reference_the experimental group_) − Ct (target gene_the control group_ − internal reference_the control group_).

**Table 1:** Primer sequence for RT-qPCR

Gene	Primer sequence (5′–3′)
BMX-ARHGAP12	F: GTGTACATTCCCACTTGGCTCG
	R: CCTGTAACTTACCCTCCCTCAG
BMX	F: GTGTTGGGGGCACTGAGTAA
	R: TCTCAGCTTGTTCCGTGCTT
ARHGAP12	F: GTGTGGTTCTCCTCCAAGGG
	R: CTTTGCTGCTGGGATGAAGC
GAPDH	F: AGAAGGCTGGGGCTCATTTG
	R: AGGGGCCATCCACAGTCTTC

*RT-qPCR* reverse transcription quantitative polymerase chain reaction, *F* forward, *R* reverse, *BMX* bone marrow X kinase (BMX), *ARHGAP* Rho GTPase activating protein, *GAPDH* glyceraldehyde-3-phosphate dehydrogenase

### Western blot assay

Cell protein and plasma membrane protein were extracted in accordance to the instructions of nuclear protein extraction kit (C500009, Sangon Biotech Co., Ltd., Shanghai, China), membrane and cytoplasmic protein extraction kit (C510005, Sangon Biotech Co., Ltd., Shanghai, China). The cells followed by trypsinization were lysed in an enhanced radio-immunoprecipitation assay (RIPA) lysis buffer (Boster Biological Engineering Company, Wuhan, Hubei, China) supplemented with a protease inhibitor. The protein concentration was determined using a bicinchoninic acid (BCA) kit (Pierce, Rockford, Waltham, MA, USA). The cell lysates were separated using a sodium dodecyl sulfate polyacrylamide gel electrophoresis (SDS-PAGE), with the isolated protein being transferred onto a polyvinylidene fluoride (PVDF) membrane set to a constant voltage of 80 V. Followed by 1-h of blocking, the membrane was incubated with diluted primary antibodies overnight at 4 °C. The primary antibodies against BMX-ARHGAP and against polyclonal antibody BMX-SH were obtained from the mice immunized as previously described [20]. The primary antibodies used included antibodies for BMX (ab207559, 1:500–1000), ARHGAP (ab74454, 1:500–1000), CD133 (ab216323, 1:1000), CD44 (ab157107, 1:2000), SOX2 (ab92494, 1:1500), JAK2 (ab200783, 1:5000), p-JAK2 (ab32101, 1:2000), STAT3 (ab68153, 1:1500), p-STAT3 (ab76315, 1:2000), Nanog (ab80892, 1:1000) and GAPDH (ab181602, 1:5000). Subsequently, either goat anti-rabbit IgG (ab205718, 1:2000–50,000) or goat anti-mouse IgG (ab6785, 1:10,000) was used as secondary antibody for 1-h incubation at 37 °C. The proteins were developed using enhanced chemiluminescence (ECL), and photographed using a SmartView Pro 2000 (UVCI-2100; Major Science, Saratoga, CA, USA). The gray values of the protein bands were analyzed by the Quantity One software. Each experiment was conducted a total of 3 times.

### Flow cytometry

After 48 h of treatment, the cells were detached with 0.25% trypsin and cell density was adjusted into 1 × 10^6^ cells/mL. A total of 1 mL cells were centrifuged at 402×*g* for 10 min, after which the obtained pellet was added with 2 mL PBS, and centrifuged again to remove the supernatant. Subsequently, the cells were resuspended into 100 µL and then incubated at 4 °C for 30 min with the addition of antibody to CD44 (11-0441, BD Biosciences, Franklin Lakes, NJ, USA), isotype control antibody for CD44 (11-4031, BD Biosciences, Franklin Lakes, NJ, USA) or antibody to CD133 (130-080-801, Miltenyi Biotec, Bergisch Gladbach, Germany), isotype control antibody for CD133 (130-092-212, Miltenyi Biotec, Bergisch Gladbach, Germany). After that, the cells were washed twice with PBS and resuspended into 100 uL cell suspension. After being filtered with 100-meshes nylon mesh, the CD44 and CD133 positive cells were sorted using flow cytometer (BD Biosciences, Franklin Lakes, NJ, USA) [21].

### Immunofluorescence assay

The transfected cells were detached, subsequently inoculated into an immunofluorescence chamber at a density of 2 × 10^5^ cells/well. Once the confluence reached approximately 90%, the cells were washed 3 times using PBS on ice. The cells were then fixed in a 4% paraformaldehyde (1 mL/well) at room temperature for 15 min. Next, the cells were permeabilized using a 0.3% Triton for 10 min. Followed by blocking with a goat serum for 30 min, the cells were incubated with primary antibodies PBS-diluted CD133 (ab19898, 1:1000) and p-STAT3 (ab32143, 1:500) at 4 °C overnight. The cells were incubated with their corresponding secondary antibody for 1 h at room temperature void of light. The cells were subsequently stained using a 4′,6-diamidino-2-phenylindole (DAPI) for 15 min in the dark. Then, the cells were mounted with a fluorescent quenching agent, followed by visualization under a fluorescence microscope.

### Cell sphere formation assay

Next, the transfected cells were inoculated into a 96-well ultra-low attachment plate at 1 × 10^4^ cells per well, followed by re-suspension in a serum-free Dulbecco's modified Eagle's medium (DMEM)-F12 containing 20 ng/mL basic fibroblast growth factor (bFGF)-β and 20 ng/mL epidermal growth factor (EGF); half of the medium was renewed every two days. After a 10-day culture, the fully formed cell spheres were observed under an inverted optical microscope (CKX41; Olympus, Shinjuku, Tokyo, Japan).

### Colony formation assay

The sterilized culture dish (100 mm) was then coated using 2 mL of 0.7% agarose (prepared using fresh DMEM) prior to beginning of the experiment. In order to examine the ability of clone formation, 0.35% agarose-cell suspension mixture was prepared with cell suspension (1 mL) mixed in 0.7% agarose solution (1 mL). The cells were then inoculated into culture dishes at a density of 1 × 10^3^ cells/10 cm^2^, with 3 parallel wells provided for each sample. When the upper agarose solidified, the cells were cultured with 2–3 mL of culture medium on the surface at 37 °C with 5% CO_2_; the medium was changed every 2–3 days. After 1 month of culture, the cell colonies (over 50 cloned cells) were observed and counted under an inverted microscope.

### GC stem cell sorting

The GC cells during the logarithmic phase were washed 3 times with PBS, followed by treatment with 0.25% trypsin into a single cell suspension. After centrifugation for 10 min, the supernatant was removed, while the cells were re-suspended using PBS. The cells (1 × 10^7^ cells/100 μL) were added with the FcR antagonists (100 μL) to block the non-specific binding. Antibodies of CD133 (ab19898, 1:300) and CD44 (ab157107, 1:300) were added into the cells for 30 min of incubation in complete darkness. After being washed with approximately 3 mL of washing buffer, the cells were centrifuged at 300×*g* for 10 min. With the removal of the supernatant, the cells were sorted in a cell strainer. The sorted cells were re-suspended into single cell suspension in the sorting buffer, while the sorted cells were identified using a flow cytometer.

### 5-Ethynyl-2′-deoxyuridine (EdU) labeling assay

EdU labeling assay was performed to evaluate the cell proliferation [22]. The transfected cells would then be seeded into a 24-well plate, each equipped with 3 parallel wells. Next, the cells in each well were cultured using EdU (10 µmol/L; C10341-1, Guangzhou RiboBio Co., Ltd., Guangzhou, Guangdong, China) for 2 h. The cells were fixed in PBS containing 4% formaldehyde at room temperature for 15 min, followed by 2 additional washes in PBS containing 3% bovine serum albumin (BSA). Next, the cells were permeabilized with PBS containing 0.5% Triton X-100 at room temperature for 20 min. Afterwards, the cells in each well were stained with 100 µL of staining solution for 30 min. The cells were stained with DAPI, followed by PBS washes. The EdU positive cells were visualized in 6–10 fields at random under a fluorescence microscope (FM-600; Pudan optical instrument Co., Ltd., Shanghai, China). The EdU labeling rate = the proportion of EdU positive cells (EdU positive cells + EdU negative cells) × 100%. The experiment was repeated three times.

### Transwell assay

Then, the Matrigel (Becton, Dickinson Co., Franklin Lakes, NJ, USA) was evenly added into the bottom of the apical chamber in the Transwell chamber and polymerized at 37 °C for 30 min. The basement membrane was hydrated prior to detection. The transfected cells were starved for 24-h in serum-free medium. The collected cells were then re-suspended with serum-free medium (1 × 10^5^ cells/mL). The medium containing 10% fetal bovine serum was added to the basolateral chamber and 100 µL of cell suspension was placed into the apical chamber. The chamber was incubated for 24 h at 37 °C in an incubator with 5% CO_2_. The cells in the apical chamber were wiped off using cotton swabs. The collected cells were fixed with a 100% methanol, and stained with toluidine blue (Sigma, St. Louis, MO, USA). The invaded cells were subsequently visualized in 5 randomly selected fields under an inverted microscope (CarlZeiss, Jena, Germany). Each experiment was conducted a total of 3 times.

### Tumor xenografts in nude mice

A total of 28 healthy nude mice (aged 4–6 weeks) were purchased from Institute of Materia Medical, Chinese Academy of Medical Sciences (Beijing, China). All mice were raised in individual cages in a specific pathogen free (SPF) animal laboratory, at 22–25 °C with a humidity of 60–65% and we permitted with free access to water and food, under a 12-h light/12-h dark cycle. Followed by one week of adaptive feeding, the nude mice were subcutaneously injected with the transfected GC cells to simulate the GC. The tumor volume and weight were measured after the nude mice were euthanatized. The expression of CD133, CD44, SOX2, and Nanog was determined by western blot assay.

### Statistical analysis

All data were analyzed by the SPSS 21.0 software (IBM Corp. Armonk, NY, USA). All data were tested for normal distribution and homogeneity of variances. The measurement data conformed to normal distribution were expressed using the mean ± standard deviation, otherwise, the skewed data with heterogeneity of variance were expressed as inter-quartile range. GC tissues and normal counterpart were compared by the paired *t*-test, while that of other data between two groups was compared by an unpaired *t*-test. The comparison of skewed data between two groups were tested using Wilcoxon rank sum test. One-way analysis of variance (ANOVA) and post hoc test were conducted for comparisons among multiple groups. Comparisons of the skewed data among multiple groups were examined using a Kruskal–Wallis H test. Data at different time points were compared by making repeated measurements with ANOVA.* p* < 0.05 was considered to be statistically significant.

## Results

### BMX-ARHGAP is highly expressed in GC tissues and cells

**Fig. 1:**
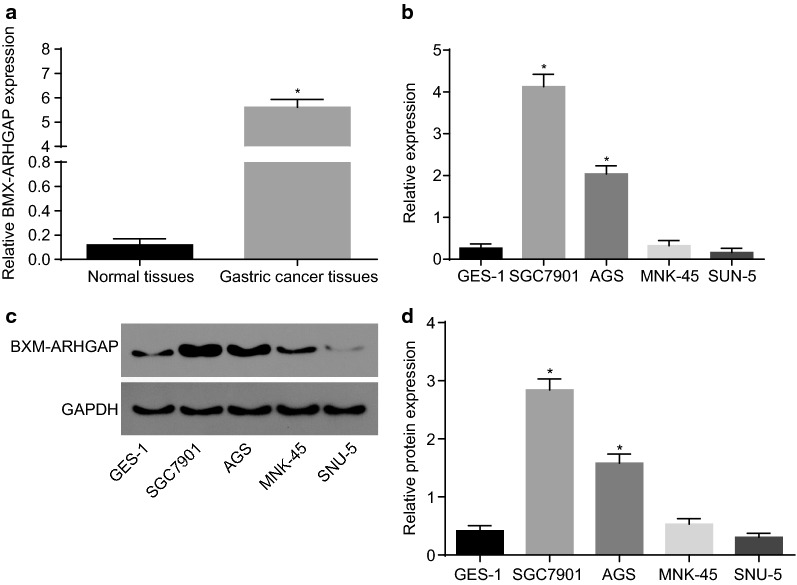
High BMX-ARHGAP expression is observed in GC tissues and cells. **a** BMX-ARHGAP expression in GC tissues and adjacent normal tissues determined by RT-qPCR; **b** the BMX-ARHGAP expression in GC cell lines (SNU-5, MNK-45, AGS and SGC7901) and normal gastric epithelial cell line (GES-1) determined by RT-qPCR; **c**, **d** the BMX-ARHGAP protein expression in GC cell lines (SNU-5, MNK-45, AGS and SGC7901) and normal gastric epithelial cell line (GES-1) measured by western blot assay. *p < 0.05 vs. the adjacent normal tissues or GES-1 cells. Enumeration data in **a** were expressed as mean ± standard deviation and compared with paired *t* test; measurement data in **b**, **d** were expressed as mean ± standard deviation and analyzed by one-way analysis of variance; each experiment was repeated three times. *BMX* bone marrow kinase on chromosome X, *ARHGAP* RhoA GTPase activating protein, *RT-qPCR* reverse transcription quantitative polymerase chain reaction, *GC* gastric cancer

In order to identify the characteristic of BMX-ARHGAP in GC, its expression in the GC tissues from 165 patients was determined by RT-qPCR. The obtained results exhibited that BMX-ARHGAP was enriched in GC tissues, but barely found in normal counterpart (Fig. 1a). Next, the BMX-ARHGAP mRNA and protein expression in GC cell lines (SNU-5, MNK-45, AGS, and SGC7901), and normal gastric epithelial cell line (GES-1) were all determined by means of RT-qPCR and western blot assay. In comparison with GES-1 cells, AGS and SGC7901 cells displayed a high mRNA and protein expression of BMX-ARHGAP (*p* < 0.05); furthermore, the SGC7901 cells had the highest BMX-ARHGAP mRNA and protein expression, thus SGC7901 cells were selected for following cell experiments (Fig. 1b–d). The aforementioned data suggested that BMX-ARHGAP was highly expressed in GC.

### BMX-ARHGAP overexpression enhances self-renewal potential of GC cells and proliferative ability of CD133^+^CD44^+^ cells

**Fig. 2:**
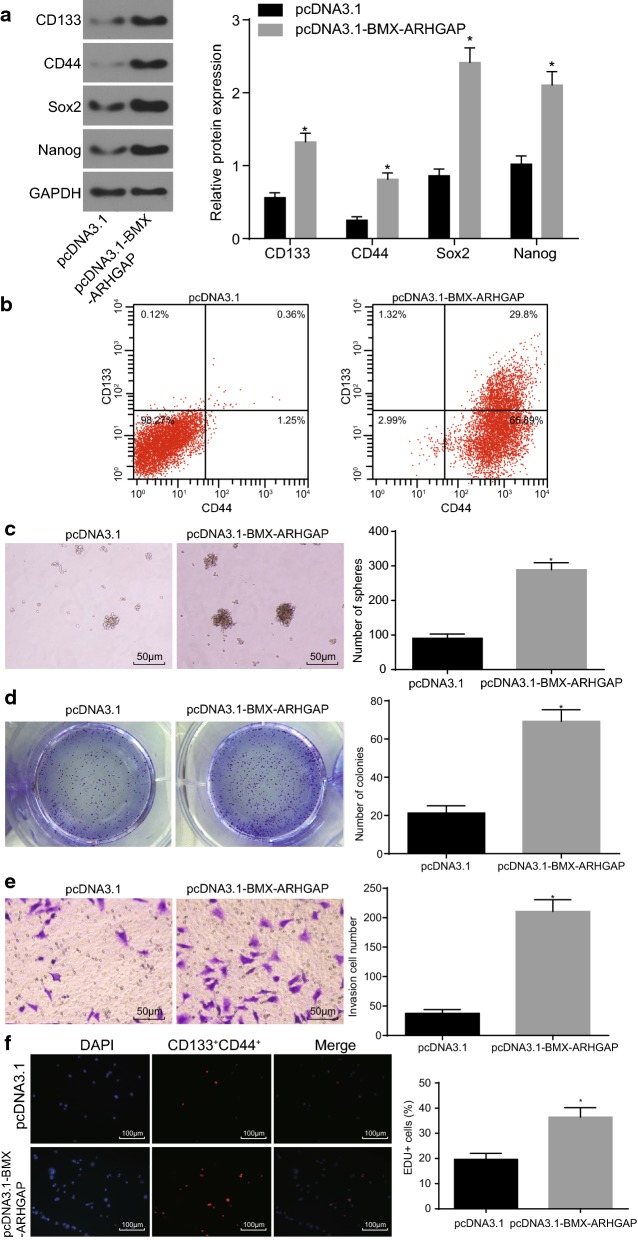
BMX-ARHGAP upregulation induces SGC7901 stem cell formation, proliferation and invasion. **a** Protein expression of CD133, CD44, SOX2 and Nanog in transfected SGC7901 cells determined by western blot assay; **b** CD133^+^CD44^+^ cells sorted by flow cytometry; **c** the number of formed cell spheres after transfection shown by cell sphere formation assay ( ×200); **d** the number of formed cell colonies after transfection shown by cell colony formation assay; **e**, the invasion ability of CD133^+^ and CD44^+^ evaluated by Transwell assay (×200); **f** proliferation ability of CD133^+^ and CD44^+^ evaluated by EdU labeling assay (×100). *p < 0.05 vs. the pcDNA3.1-transfected cells. Measurement data were expressed as mean ± standard deviation and analyzed by independent *t*-test; each experiment was repeated three times. *BMX* bone marrow kinase on chromosome X, *ARHGAP* RhoA GTPase activating protein

BMX-ARHGAP expression in SGC7901 cells were elevated using pcDNA3.1-BMX-ARHGAP to examine the effects BMX-ARHGAP had on the SGC7901 stem cell properties. The expression of the stem cell surface markers (CD133 and CD44) and the pluripotency transcriptional factor markers (SOX2 and Nanog) were all measured by western blot assay. In comparison with the negative control, the SGC7901 cells producing an overexpression of BMX-ARHGAP displayed a remarkably increased expression of CD133, CD44, SOX2, and Nanog (*p* < 0.05) (Fig. 2a). The results of flow cytometry revealed that the percentage of CD133^+^CD44^+^ cells was detected to be higher in the SGC7901 cells overexpressing BMX-ARHGAP as opposed to that in the pcDNA3.1-transfected SGC7901 cells (Fig. 2b). Afterwards, the results of cell sphere formation and colony formation assays revealed that the SGC7901 cells overexpressing BMX-ARHGAP had remarkably elevated numbers of formed cell spheres and cell colonies as opposed to the pcDNA3.1-transfected SGC7901 cells (*p* < 0.05) (Fig. 2c, d). Further sorting of CD133^+^CD44^+^ SGC7901 cells was completed by conducting flow cytometry. The sorted CD133^+^CD44^+^ cells were transfected with pcDNA3.1-BMX-ARHGAP. Transwell and EdU labeling assays were conducted to assess the invasion and proliferation abilities of the transfected CD133^+^CD44^+^ cells with the purpose of identifying the role BMX-ARHGAP in stem cell properties. The obtained results showed that the transfection of pcDNA3.1-BMX-ARHGAP led to an enhanced proliferation and invasion of CD133^+^CD44^+^ cells (*p* < 0.05) (Fig. 2e, f). The aforementioned results demonstrated that the overexpression of BMX-ARHGAP promotes self-renewal potential of SGC7901 cells and facilitates CD133^+^CD44^+^ cell proliferation and invasion.

### BMX-ARHGAP overexpression increases BMX-SH2 protein expression via 5′-UTR of ARHGAP12 mRNA

**Fig. 3:**
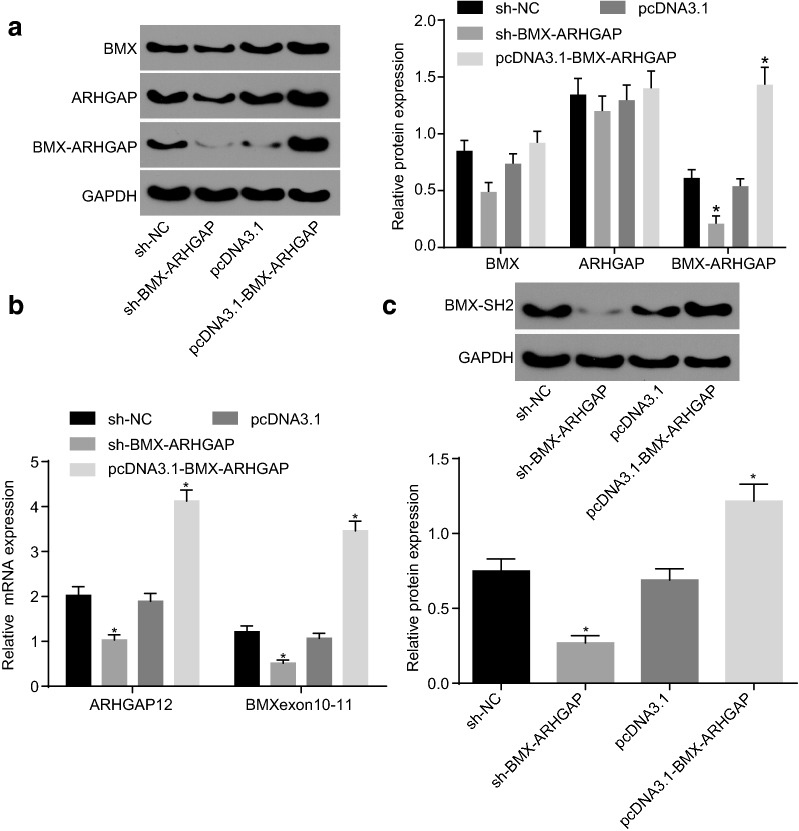
BMX-ARHGAP overexpression elevates the BMX-SH2 protein expression via ARHGAP 5′UTR. **a** Protein expression of BXM, ARHGAP and BMX-ARHGAP in transfected SGC7901 cells, as measured by western blot assay; **b** BMX exon 10–11 and ARHGAP12 5′UTR mRNA expression after transfection, as determined by means of RT-qPCR; **c** protein expression of BMX-SH2 after transfection, as measured by western blot assay; *p < 0.05 vs. the cells transfected with pcDNA3.1 or sh-NC. Measurement data were expressed as mean ± standard deviation and analyzed by one-way analysis of variance; each experiment was repeated three times. *BMX* bone marrow kinase on chromosome X, *ARHGAP* RhoA GTPase activating protein, *RT-qPCR* reverse transcription quantitative polymerase chain reaction

The protein expression of BMX, ARHGAP, and BMX-ARHGAP was measured by western blot assay after transfection. The western blot assay results showed that the BMX-ARHGAP protein expression profoundly decreased in the cells expressing sh-BMX-ARHGAP, or remarkably elevated in the cells expressing pcDNA3.1-BMX-ARHGAP (all *p* < 0.05). However, no significant differences were observed regarding to BMX and ARHGAP protein expression among the four groups (*p* > 0.05) (Fig. 3a). The expression of BMX and ARHGAP12 mRNA was determined by conducting a RT-qPCR, the results of which showed that the ARHGAP12 5′UTR and BMX exon 10–11 mRNA expression dramatically decreased in the cells expressing sh-BMX-ARHGAP, while elevation was detected in the cells expressing pcDNA3.1-BMX-ARHGAP (all *p* < 0.05) (Fig. 3b). BMX exon 10–11 encodes SH2-domain protein. Hence, the expression of SH2-domain protein (BMX-SH2) was measured by western blot assay. The results exhibited that BMX-SH2 protein expression markedly reduced due to the transfection of sh-BMX-ARHGAP, while elevated by transfection of pcDNA3.1-BMX-ARHGAP (all *p* < 0.05) (Fig. 3c). Based on those results, BMX-ARHGAP upregulation is suggested to increase the BMX-SH2 protein expression via ARHGAP 5′UTR.

### BMX-ARHGAP activates the JAK/STAT3 signaling pathway via increasing BMX-SH2 protein expression

**Fig. 4:**
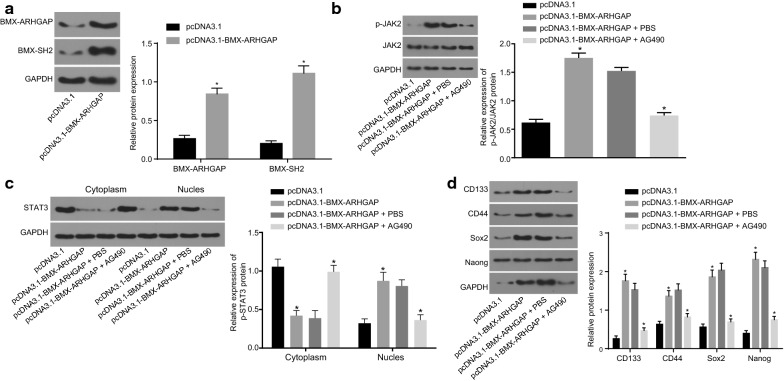
BMX-ARHGAP overexpression induces the activation of JAK/STAT3 signaling pathway by upregulating BMX-SH2. **a** Protein expression of BMX-ARHGAP and BMX-SH2 in transfected SGC7901 cells measured by western blot assay; **b** the protein expression of JAK2 and STAT3 and their phosphorylation extents after transfection measured by western blot assay; **c** the extent of STAT3 phosphorylation in the nucleus and cytoplasm measured by western blot assay; *C* cytoplasm, *N* nucleus, *D* protein expression of CD133, CD44, SOX2 and Nanog in transfected SGC7901 cells determined by western blot assay; *p < 0.05 vs. the cells transfected with pcDNA3.1 or treated with pcDNA3.1-BMX-ARHGAP and PBS. Measurement data were expressed as mean ± standard deviation; data in **a** were analyzed by independent *t* test and those in **b**–**d** were analyzed by one-way analysis of variance; each experiment was repeated three times. *BMX* bone marrow kinase on chromosome X, *ARHGAP* RhoA GTPase activating protein, *SH2* Src homology region 2, *PBS* phosphate buffer saline, *JAK2* Janus kinase 2, *STAT3* signal transducer and activator of transcription 3

The protein expression of BMX-ARHGAP and BMX-SH2 was measured by western blot assay. It was shown that the transfection of pcDNA3.1-BMX-ARHGAP resulted in an elevated protein expression of BMX-ARHGAP and BMX-SH2 (*p* < 0.05) (Fig. 4a). In order to analyze the regulatory effects that BMX-ARHGAP had on the JAK/STAT3 signaling pathway, the protein expression of JAK2 and STAT3, and the extents of AK2 and STAT3 phosphorylation were examined by specific antibodies. The results obtained presented that the extents of JAK2 and STAT3 phosphorylation were significantly upregulated in the cells overexpressing BMX-ARHGAP, while the treatment of AG490 reversed the aforementioned upregulation (*p* < 0.05). The ratios of p-JAK2/JAK2 and p-STAT3/STAT3 in the cells overexpressing BMX-ARHGAP were remarkably higher than in the cells transfected with pcDNA3.1; whereas, those were evidently reduced by treatment with pcDNA3.1-BMX-ARHGAP and AG490, in comparison to the cells treated with pcDNA3.1-BMX-ARHGAP and PBS (*p* < 0.05; Fig. 4b). The western blot assay assessed the extent of the STAT3 phosphorylation in the nucleus, showing that the expression of phosphorylated STAT3 in the nucleus of the cells overexpressing BMX-ARHGAP was remarkably increased; whereas, that was reduced in the presence of additional AG490 treatment (*p* < 0.05) (Fig. 4c). Meanwhile, the protein expression of CD133, CD44, SOX2, and Nanog determined by western blot assay was found to be profoundly elevated by the transfection of pcDNA3.1-BMX-ARHGAP, but this elevation was reversed due to the additional treatment of AG490 (*p* < 0.05) (Fig. 4d). Hence, those results suggested a regulatory network that BMX-ARHGAP induces the activation of the JAK/STAT3 signaling pathway by upregulating BMX-SH2.

### BMX-ARHGAP maintains the tumorigenic potential of GC cells though the JAK/STAT3 signaling pathway activation

**Fig. 5:**
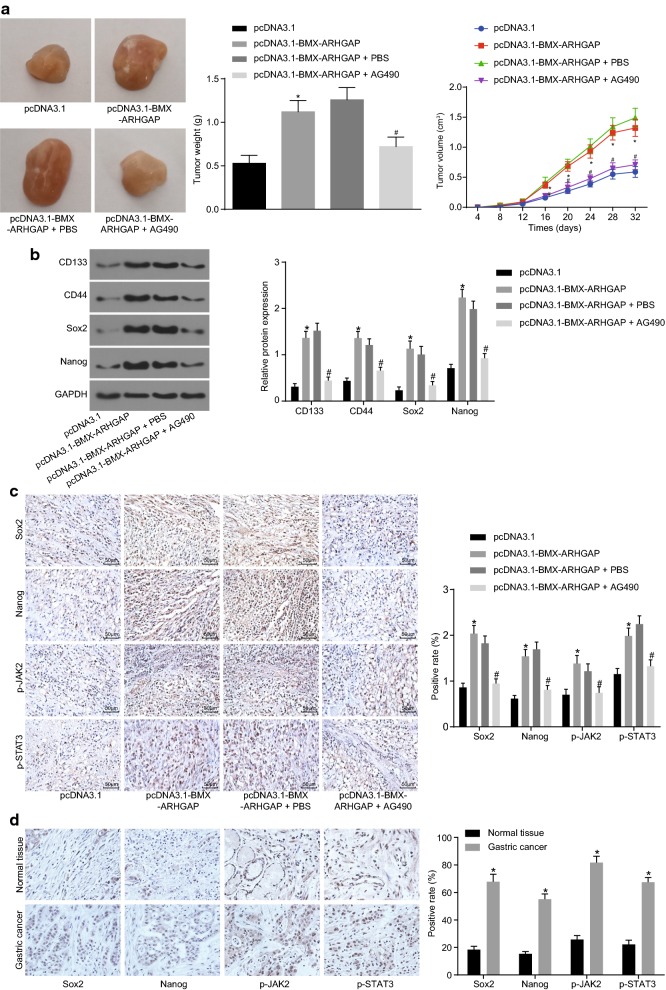
BMX-ARHGAP overexpression enhances tumorigenicity of SGC7901 cells in nude mice. **a** Tumor volume and weight in nude mice injected with transfected SGC7901 cells; **b** protein expression of CD133, CD44, SOX2 and Nanog in the tumor tissues of nude mice injected with transfected GC cells, as determined by western blot assay; **c** protein expression of SOX2 and Nanog together with the extents of JAK2 and STAT3 phosphorylation in the tumor tissues of nude mice injected with transfected SGC7901 cells, as measured by immunohistochemical staining (×200); **d** immunohistochemical staining of Sox2, Nanog, p-JAK2, p-STAT3 positive expression in GC and adjacent normal tissues (×400); *p < 0.05 vs. the injection of pcDNA3.1-transfected cells; ^#^*p* < 0.05 vs. the treatment of pcDNA3.1-BMX-ARHGAP and PBS. Enumeration data in **a** were expressed as mean ± deviation and analyzed by one-way and repeated measurement analysis of variance; measurement data in **b**, **c** were analyzed by one-way analysis of variance; each experiment was repeated three times. *BMX* bone marrow kinase on chromosome X, *ARHGAP* RhoA GTPase activating protein, *PBS* phosphate buffer saline, *JAK2* Janus kinase 2, *STAT3* signal transducer and activator of transcription 3

With the aim to explore the role of BMX-ARHGAP in tumor formation and growth, the stable transfected SGC7901 cells were delivered into the nude mice. The measurement results of tumor volume and weight revealed that they were elevated due to the transfection of pcDNA3.1-BMX-ARHGAP, while this enhancement was reversed by the treatment of AG490 (*p* < 0.05) (Fig. 5a). As shown by the western blot assay results regarding to stem cell surface markers (CD133 and CD44) and pluripotency markers (SOX2 and Nanog), the protein expression of CD133, CD44, SOX2, and Nanog was increased in response to the transfection of pcDNA3.1-BMX-ARHGAP (*p* < 0.05). However, the expression of those aforementioned proteins was lowered due to the treatment of AG490 in the cells overexpressing BMX-ARHGAP (*p* < 0.05) (Fig. 5b). As shown by an immunohistochemical staining, either yellow or brown stained cells were considered as positive (the positive-stained proteins located in the nucleus as indicated by the arrow). The protein expression of SOX2 and Nanog and the extent of JAK2 and STAT3 phosphorylation were prominently increased in the cells overexpressing BMX-ARHGAP, as opposed to those in the pcDNA3.1-transfected cells; treatment with AG490 caused a reduction in the expression and phosphorylation extent in the cells overexpressing BMX-ARHGAP (*p* < 0.05) (Fig. 5c). Furthermore, the protein expression for Sox2 and Nanog and the extent of JAK2 and STAT3 phosphorylation were measured by an immunohistochemical staining in the coupling clinical GC tissues and adjacent normal tissues. The positive expression was mainly observed by either yellow or brown granules, which were directly located in the nucleus. The protein expression of Sox2 and Nanog and the extent of JAK2 and STAT3 phosphorylation in GC tissues were higher than in adjacent normal tissues (*p* < 0.05; Fig. 5d). These results supported that BMX-ARHGAP overexpression enhances tumorigenicity of SGC7901 cells through the activation of the JAK/STAT3 signaling pathway.

## Discussion

Gene fusions remarkably alter gene structures leading to mutated protein products with an aberrant molecular function. They are usually involved in tumor progression through modulation of tumorigenic potential of the cancer stem cells [23]. The present study identified the high expression of BMX-ARHGAP fusion in GC. The gain-of-function experiment revealed that BMX-ARHGAP promoted self-renewal potential of SGC7901 cells and facilitated proliferation and invasion of CD133- and CD44-expressing cells in GC. To that point, our findings suggested that BMX-ARHGAP overexpression enhances self-renewal potential of GC cells and proliferative potential of stem cells in vitro and tumorigenicity in vivo through activation of the SH2-domain-mediated JAK/STAT3 signaling pathway.

Consistent with the previous finding [10], BMX-ARHGAP was expressed remarkably in GC tissues and cells. At first, the gain-of-function experiment revealed that BMX-ARHGAP overexpression increased expression of CD133, CD44, SOX2 and Nanog, and accelerated proliferation and invasion of GC cells and CD133, CD44 positive cells, indicating a promotive effect on GC stem cell properties. CD133 and CD44 are two stem cell markers, while SOX2 and Nanog are two transcription factors involved in the maintenance of cancer stem-like cell properties [24]. Cancer stem cells could proliferate and differentiate into heterogenic tumor cells, conferring to the tumor cells diversity and leading to drug resistance [25]. Either BMX or ARHGAP alone has been determined to drive tumor development. For example, BMX is considered to be an essential factor for the maintenance of glioma stem cell-derived pericytes, while the inhibition of BMX is known to improve chemotherapeutic efficacy [26]. BMX contributes to the castration resistance in prostate cancer by positively mediating the activities of multiple receptor tyrosine kinases (RTK) through inducing the generation of phosphotyrosine-tyrosine (pYY) phosphorylation in their activation loop [27]. A previous study reported by Dong et al*.* has suggested an oncogenic role of CLDN18-ARHGAP26/6 fusion, the fusion status of which is an independent predictive indicator for distant organ metastases in diffuse-type GC [28]. Therefore, BMX and ARHGAP play wide oncogenic roles on multiple cancers progression.

In this study, our results demonstrated that the inhibition of the JAK/STAT3 signaling pathway reversed the stimulating role of BMX-ARHGAP in GC stem cells, effectively confirming the vital role of JAK/STAT3 in BMX-ARHGAP regulation. BMX-ARHGAP activates the JAK/STAT3 signaling pathway, as evidenced by an enhanced JAK2 and STAT3 phosphorylation. Mechanistically, BMX bypasses the suppressor of cytokine signaling 3 (SOCS3)-mediated inhibition of JAK2, whereas targeting of the BMX dampens the JAK2-mediated STAT3 activation, underlying a new molecular basis in which to specifically eliminate the glioma stem cells [29]. Additionally, BMX knockdown could block the STAT3 activation, while inhibiting the glioblastoma stem cell transcription factors to disrupt the tumorigenicity of glioblastoma stem cells [30]. The activated JAK/STAT3 signaling pathway was closely associated with the invasion and metastasis of GC [31]. Conversely, an increase of GC cell migration and invasion could be attenuated by the blocking the JAK/STAT3 signaling pathway [32]. Although the regulation of this pathway in GC stem cell properties has not been well-understood, this pathway is a master regulator of cancer stem cell properties in other cancers such as hepatocellular carcinoma [33]. Hence, it is deemed rationale to speculate that BMX-ARHGAP fusion is able to activate the JAK/STAT3 signaling pathway in the maintenance of GC stem cells.

The study suggested a potential regulatory network by which BMX-ARHGAP induced the activation of the JAK/STAT3 signaling pathway by upregulating BMX-SH2. Over 50 secreted glycoproteins modulate hematopoiesis and the immune response via the JAK/STAT pathway, transferring the extracellular ligands signal through a phosphotyrosine-based intracellular signaling cascade induced by kinases [34]. The SH2 domain bind phosphorylated tyrosine, thereby regulating the activity of those kinases. For instance, STAT1 molecule modulates SH2 domain to induce the IFN-γ signaling through increasing of STAT1 phosphorylation [35]. STAT activation was induced by phosphorylation at the STAT SH2 domain [36]. Interestingly, SH-2 domain-containing phosphatase 1 (SHP1) and SH-2 domain-containing phosphatase 2 (SHP2) have been demonstrated to have induced STAT3 phosphorylation, thereby controlling STAT3 signaling [37]. In this study, the results obtained indicated that BMX-ARHGAP increased the BMX-SH2 protein expression through ARHGAP 5′UTR, subsequently activating JAK2 and STAT3 phosphorylation. In lung cancer, co-repression of STAT3 and ARHGAP35 signaling is conducive for cancer treatment [38]. SIRT1 restraints the progression of GC via the modulation of ARHGAP5 expression, representing a novel mechanism of SIRT1 as a tumor suppressor [39]. In this regard, a potential conclusion can be drawn that BMX-ARHGAP/SH2 domain/JAK/STAT3 axis modulates the GC development.

## Conclusions

**Fig. 6:**
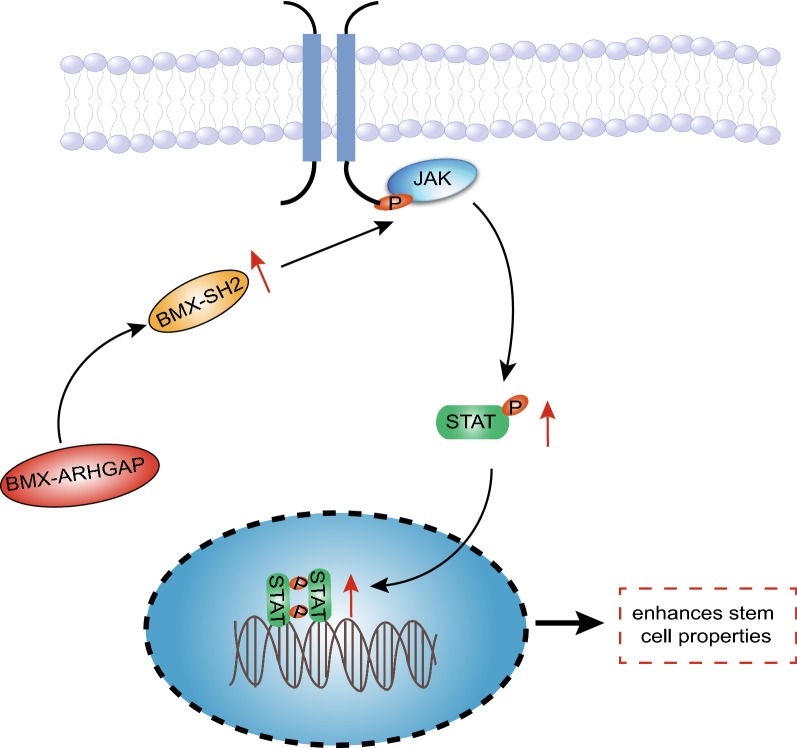
The regulatory mechanism of BMX-ARHGAP in the properties of GC stem cells. BMX-ARHGAP fusion gene activates JAK/STAT3 signaling pathway through BMX-SH2 protein to maintain the stemness of GC stem cells, thereby inducing the tumorigenicity of GC stem cells

To summarize all that we have gathered, the aforementioned data supported a conclusion that BMX-ARHGAP induces the activation of the JAK/STAT3 signaling pathway by upregulating BMX-SH2, underlying a novel mechanism of the oncogenic BMX-ARHGAP fusion in GC (Fig. 6). Interestingly enough, SOCS3 is known to mediate the JAK/STAT3 signaling pathway to regulate cell proliferation, differentiation, and apoptosis in lung cancer [40]. However, due to limited time, the potential involvement of SOCS3 in this regulation was not thoroughly investigated. Due to the lack of investigation, we plan on exploring SOCS3 as a future topic for the discovery of new therapeutic options for gastric cancer.

## Data Availability

The datasets generated and/or analysed during the current study are available from the corresponding author on reasonable request.

## Abbreviations

CSCs: cancer stem cells
GC: gastric cancer
BMX: bone marrow kinase on chromosome X
ARHGAP: RhoA GTPase activating protein
STAT: signal transducer and activator of transcription
JAK: Janus kinase
SH2: Src-homology 2
IgG: immunoglobulin G
HRP: horseradish peroxidase
DAB: diaminobenzidine
ATCC: American Type Culture Collection
MOI: multiplicity of infection
GAPDH: glyceraldehyde-3-phosphate dehydrogenase
RIPA: radio-immunoprecipitation assay
BCA: bicinchoninic acid
SDS-PAGE: sodium dodecyl sulfate polyacrylamide gel electrophoresis
PVDF: polyvinylidene fluoride
ECL: enhanced chemiluminescence
BSA: bovine serum albumin
SPF: specific pathogen free
ANOVA: analysis of variance
